# Select Small Core Structure Carbamates Exhibit High Contact Toxicity to “Carbamate-Resistant” Strain Malaria Mosquitoes, *Anopheles gambiae* (Akron)

**DOI:** 10.1371/journal.pone.0046712

**Published:** 2012-10-01

**Authors:** Dawn M. Wong, Jianyong Li, Qiao-Hong Chen, Qian Han, James M. Mutunga, Ania Wysinski, Troy D. Anderson, Haizhen Ding, Tiffany L. Carpenetti, Astha Verma, Rafique Islam, Sally L. Paulson, Polo C.-H. Lam, Maxim Totrov, Jeffrey R. Bloomquist, Paul R. Carlier

**Affiliations:** 1 Department of Chemistry, Virginia Tech, Blacksburg, Virginia, United States of America; 2 Department of Biochemistry, Virginia Tech, Blacksburg, Virginia, United States of America; 3 Department of Entomology, Virginia Tech, Blacksburg, Virginia, United States of America; 4 Department of Entomology and Nematology, Emerging Pathogens Institute, University of Florida, Gainesville, Florida, United States of America; 5 Molsoft LLC, San Diego, California, United States of America; Weizmann Institute of Science, Israel

## Abstract

Acetylcholinesterase (AChE) is a proven target for control of the malaria mosquito (*Anopheles gambiae*). Unfortunately, a single amino acid mutation (G119S) in *An. gambiae* AChE-1 (*Ag*AChE) confers resistance to the AChE inhibitors currently approved by the World Health Organization for indoor residual spraying. In this report, we describe several carbamate inhibitors that potently inhibit G119S *Ag*AChE and that are contact-toxic to carbamate-resistant *An. gambiae*. PCR-RFLP analysis was used to confirm that carbamate-susceptible G3 and carbamate-resistant Akron strains of *An. gambiae* carry wild-type (WT) and G119S AChE, respectively. G119S *Ag*AChE was expressed and purified for the first time, and was shown to have only 3% of the turnover number (*k*_cat_) of the WT enzyme. Twelve carbamates were then assayed for inhibition of these enzymes. High resistance ratios (>2,500-fold) were observed for carbamates bearing a benzene ring core, consistent with the carbamate-resistant phenotype of the G119S enzyme. Interestingly, resistance ratios for two oxime methylcarbamates, and for five pyrazol-4-yl methylcarbamates were found to be much lower (4- to 65-fold). The toxicities of these carbamates to live G3 and Akron strain *An. gambiae* were determined. As expected from the enzyme resistance ratios, carbamates bearing a benzene ring core showed low toxicity to Akron strain *An. gambiae* (LC_50_>5,000 μg/mL). However, one oxime methylcarbamate (aldicarb) and five pyrazol-4-yl methylcarbamates (**4a**–**e**) showed good to excellent toxicity to the Akron strain (LC_50_ = 32–650 μg/mL). These results suggest that appropriately functionalized “small-core” carbamates could function as a resistance-breaking anticholinesterase insecticides against the malaria mosquito.

## Introduction

Malaria presents an enormous burden in sub-Saharan Africa, killing nearly 700,000 people each year and sickening hundreds of millions more [1], [2], [3]. Fortunately, control of the disease-transmitting mosquito, *Anopheles gambiae* is a proven strategy to reduce malaria transmission [2], [4]. To date, only two biological targets have been used to control adult mosquitoes [5]: acetylcholinesterase (AChE, EC 3.1.1.7) and the voltage-gated sodium ion channel [6], [7], [8]. At present, the World Health Organization Pesticide Evaluation Scheme (WHOPES, http://www.who.int/whopes/en/) has approved five insecticidal AChE inhibitors for indoor residual spraying (IRS), but none have been approved for use on insecticide treated nets (ITNs). Instead, ITNs are impregnated with pyrethroid modulators of the voltage-gated sodium ion channel. However, emerging pyrethroid-resistant strains of *An. gambiae* put this malaria control strategy at risk [9], [10].

One way to combat this growing threat of pyrethroid resistance would be to develop new anticholinesterase-based ITNs [11], [12]. AChE rapidly hydrolyzes the neurotransmitter acetylcholine at cholinergic synapses in the central nervous system, terminating cholinergic synaptic transmission [13]. Although mosquitoes in general carry two AChE genes, *ace*-*1* and *ace-2* (encoding AChE-1 and AChE-2 proteins respectively) [14], [15], [16], in *An. gambiae*, it appears that *Ag*AChE-1 (henceforth *Ag*AChE) is primarily responsible for the nervous system cholinesterase activity [14], [17], [18]. Consequently, efforts have been made to develop safe anticholinesterase insecticides that feature high selectivity for inhibition of *Ag*AChE over human AChE (*h*AChE) [19], [20], [21], [22]. However another challenge looms: a single amino acid mutation of *Ag*AChE, identified as G119S, confers target site resistance in *An. gambiae* [17], [23], [24]. Such resistant *An. gambiae* have emerged as a consequence of the widespread use of anticholinesterase agricultural pesticides [25], [26]. This development therefore jeopardizes not only present IRS-based mosquito control efforts, but also any future anticholinesterase ITN-based strategy.

In this paper we identify a class of carbamates that show good contact toxicity to Akron strain *An. gambiae*, that we demonstrate by genetic analysis to carry the G119S mutation. Detailed kinetic characterization of recombinant wild-type (WT) and G119S *Ag*AChE is provided, and compared to that of carbamate-susceptible wild-type G3 strain and carbamate-resistant Akron *An. gambiae*. Within the set of inhibitors studied, G3 and Akron strain toxicological outcomes largely correlate to the kinetics of inhibition of WT and G119S *Ag*AChE. From these studies we conclude that small core structures are a key requirement for potent inhibition of G119S *Ag*AChE, and consequent toxicity towards *An. gambiae* carrying the G119S mutation.

## Results

### Confirmation of the Carbamate-resistant Genotype in Akron Strain An. gambiae

**Figure 1:**
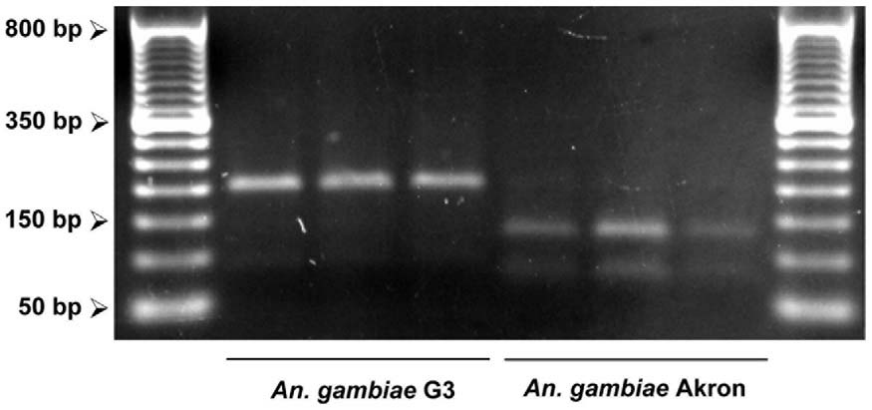
PCR amplification of G119/S119 region in single individuals of *An. gambiae* susceptible (G3) and resistant (Akron) strains. Genomic DNA amplification with Moustdir1 and Moustrev1 degenerate primers produce a 194 bp fragment, which is undigested by *Alu*I for susceptible mosquitoes (G3 strain) and digested into two fragments (122 and 72 bp) for homozygous resistant mosquitoes (Akron strain).

**Figure 2:**
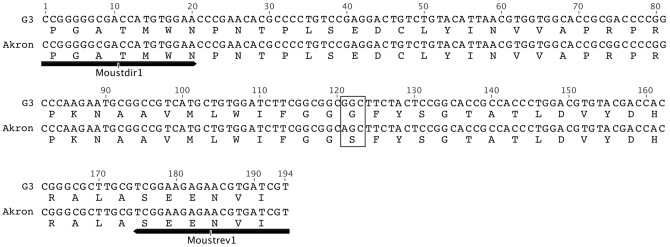
Nucleotide and deduced amino acid sequences of the *ace*-*1* amplicons of susceptible (G3) and resistant (Akron) *An. gambiae*. The alignment of DNA sequences illustrates the presence of the AG|CT *Alu*I restriction site in the Akron *ace*-*1* amplicon (194 bp) spanning the 119 (G/S) and 120 (F) codons of the *Ag*AChE-1 amino acid sequence; the 119 codon is marked with a rectangle. The arrows indicate the position of the degenerate primers (Moustdir1 and Moustrev1) used for the PCR amplification and sequencing of genomic DNA. Nucleotide numbers in the amplicon are provided above the G3 sequence.

To confirm the presence of the G119S mutation in the *ace*-*1* gene of Akron strain *An. gambiae*, we adopted the general approach for *ace*-*1* genotyping as described by Weill et al. [23], with slight modifications. The two degenerate primers Moustdir1 and Moustrev1, located in the third coding exon of the *ace*-*1* gene, allowed for the amplification of a 194 bp DNA fragment in both susceptible and resistant mosquitoes. As shown in Figure 1, the amplicon derived from the wild-type G3 strain *An. gambiae* was not digested, since it lacks the *Alu*I restriction site and thus is unaffected by treatment with the restriction enzyme. In contrast, the Akron amplicon was digested by *Alu*I, producing 122 bp and 72 bp fragments similar to that previously described in Yao resistant strain *An. gambiae* by Weill et al. [23]. For further confirmation, DNA sequencing of the *ace*-*1* amplicons for the susceptible and resistant mosquitoes was performed and demonstrated the *Alu*I restriction site at the 119 codon of Akron strain (Figure 2). In addition to confirming the G119S mutation in the *ace*-*1* gene of Akron strain *An. gambiae*, Figure 2 also demonstrates the A to G substitution at base 75 in Akron strain, as had been seen in Yao strain *An. gambiae* [23]. Thus G3 strain *An. gambiae* are homozygous susceptible, and Akron strain are homozygous resistant.

### Expression, Purification, and Characterization of WT and G119S An. gambiae AChE (AgAChE)

**Figure 3:**
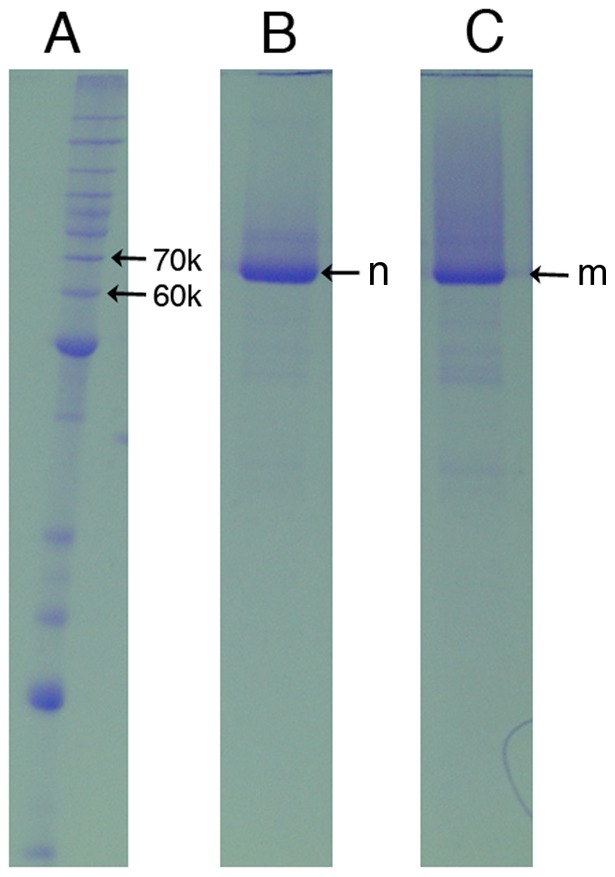
Electrophoretic analysis (SDS-PAGE) of the purified r*Ag*AChE (WT and G119S). A: Protein standard (Da); B: r*Ag*AChE-WT (“n”); C: r*Ag*AChE-G119S mutant (“m”).

Recombinant catalytic domain constructs of the WT and G119S *Ag*AChE were expressed and purified as described below in Methods. The purified catalytic domain constructs r*Ag*AChE-WT and r*Ag*AChE-G119S were investigated by SDS-PAGE analysis, and were shown to have high purity and apparent molecular masses between 60 and 70 kDa (Figure 3), close to the calculated molecular masses of the enzyme catalytic subunit constructs (64.1 kDa, Text S1). Enzymatic activities of both proteins were measured using the Ellman method [27] at pH values from 6 to 10, and as expected from previous reports on *Ag*AChE-WT [28] and native electric eel AChE [29], these studies demonstrated bell-shaped curves with maximum activity near pH 8 (Text S1). Stability of recombinant enzyme activity was assessed at 23±1°C (pH 7.7): r*Ag*AChE-WT and r*Ag*AChE-G119S have inactivation *t*_1/2_ values of 2,700±800 and 98±5 min respectively. Under these conditions recombinant *h*AChE (r*h*AChE) gave no measurable loss of activity over two hours.

The WT and G119S recombinant *Ag*AChE enzymes and r*h*AChE were characterized for catalysis of acetylthiocholine (ATCh) hydrolysis (Table 1; Michaelis-Menten plots in Text S1). Enzyme velocities were measured at ATCh concentrations up to 2 mM for *h*AChE and r*Ag*AChE-G119S and up to 1 mM for r*Ag*AChE-WT. No attempt was made to detect substrate inhibition or activation at higher substrate concentrations. The homogenates of G3 and Akron strain *An. gambiae* were also assayed in the same manner. As demonstrated above G3 is a WT carbamate-susceptible strain, and Akron carries the G119S *ace*-*1* mutation and has a carbamate-resistant phenotype. Good correspondence was seen between the *K*_m_ values of r*Ag*AChE-WT and *An. gambiae* G3 homogenate, consistent with the proposal that the major ATCh-hydrolyzing enzyme in G3 *An. gambiae* is encoded by the WT *ace*-*1* gene (Table 1). Similarly, good correspondence was seen in the *K*_m_ values of recombinant G119S *Ag*AChE and Akron homogenate, as expected. For both purified recombinant enzymes, and for homogenates, the *K*_m_ value of the G119S enzyme is 2-fold higher than that of the WT protein, suggesting slight steric hindrance of binding of substrate in the more crowded G119S active site. With regard to specific activity, we measured 2,500±100 U/mg for r*Ag*AChE-WT, and only 67±9 U/mg for the recombinant G119S mutant. In terms of *k*_cat_, a 34-fold reduction is seen in the G119S mutant. Thus the catalytic power of the enzyme is dramatically reduced in the G119S mutant. We also determined total AChE activity in the homogenates of G3 and Akron strain *An. gambiae*. For each strain, four groups of five female mosquitoes (5 days old) were weighed and homogenized to determine the total AChE activity (U) per mg mosquito. For G3 strain 0.023±0.001 U/mg mosquito was measured; for Akron strain 0.005±0.001 U/mg mosquito was measured. Thus, Akron strain mosquitoes have only 22% of the AChE catalytic activity of G3 strain mosquitoes, on a weight basis. Finally, to benchmark our methods, we determined the specific activity of the commercial *h*AChE. The value we obtained (4,100 U/mg) is higher than that quoted by Sigma, but is lower than the 6,000 U/mg value reported in the literature [30], [31], [32].

**Table 1:** Kinetic parameters (23±1°C, pH 7.7) of r*Ag*AChE (WT & G119S) and r*h*AChE, and *K*_m_ values for the ATCh-hydrolyzing enzyme in *An. gambiae* homogenate (G3 and Akron).

	*K*_m_	*V*_max_	*k*_cat_	*k*_cat_/K_m_	Specific Activity
enzyme	(μM)	(U/mg)^a^	(min^−1^)^b^	(min^−1^ mM^−1^)	(U/mg)^c^
r*Ag*AChE-WT	53.8±1.4	2,700±100	1.8±0.1 * 10^5^	3.3±0.2 * 10^6^	2,500±100
*An. gambiae* G3 homogenate	47.3±2.1				
r*Ag*AChE-G119S	128±3	83±11	5.3±0.7 * 10^3^	0.042±0.007 * 10^6^	67±9
*An. gambiae* Akron homogenate	109±8				
r*h*AChE	201±11	5,600±100	3.6±0.1 * 10^5^	1.8±0.1 * 10^6^	4,100±100

a Enzyme velocity at saturating ATCh concentrations; 1 unit (U)  = 1 μmol ATCh substrate processed per minute (μmol min^−1^). Protein concentrations were determined using the Thermo Scientific Micro BCA Protein Assay Kit 23235 (see Materials and Methods). ^b^Turnover numbers (*k*_cat_) were determined based on *V*_max_ and the calculated molecular mass of the enzyme catalytic subunits (see Materials and Methods). ^c^Specific activity determined at [ATCh]  = 0.50 mM, according to convention.

### Inhibition of WT and G119S AgAChE by Aryl and Oxime Methylcarbamate Insecticides

**Figure 4:**
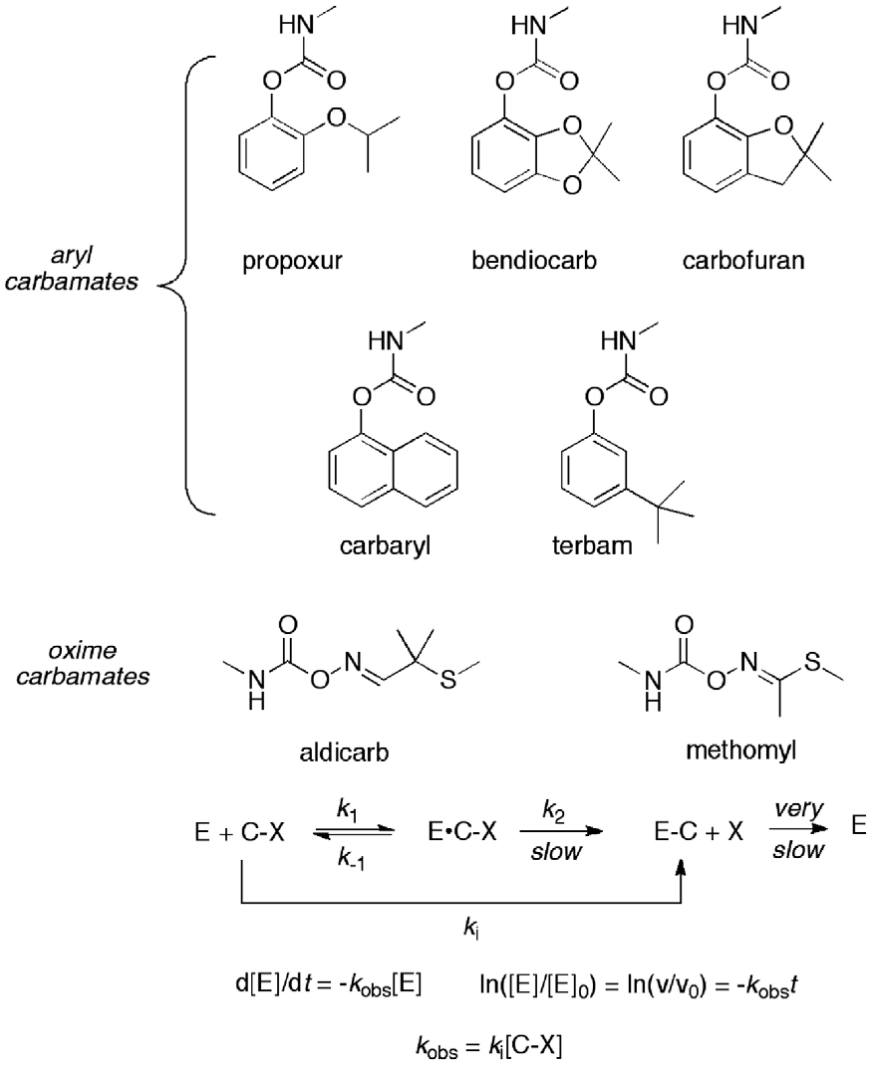
Structures of aryl and oxime carbamates studied, and scheme for carbamate (C–X) inhibition. Kinetic model describes determination of *k*_obs_ by observing first-order loss of enzyme activity (v/v_0_) at a fixed inhibitor concentration [I].

**Figure 5:**
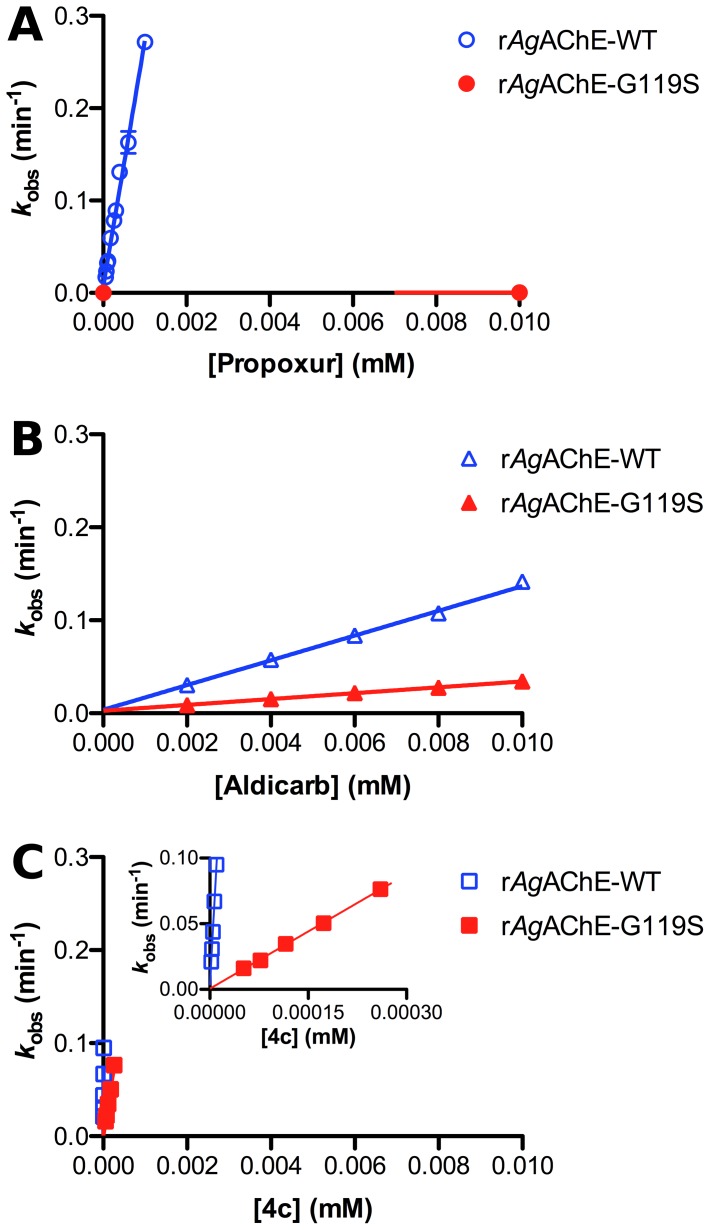
Plots of *k*_obs_ vs [carbamate] at both r*Ag*AChE-WT and r*Ag*AChE-G119S for three inhibitors. A) propoxur; B) aldicarb; C) **4c**. Second-order rate constants for inactivation *k*_i_ (mM^−1^ min^−1^) derive from the slope of each line. For clarity the data for **4c** are also plotted on expanded axes (inset).

To confirm the carbamate-resistant phenotype expected for the G119S enzyme and Akron strain *An. gambiae*, we measured the kinetics of inhibition of the various enzyme sources with a series of aryl and oxime methylcarbamate inhibitors (Figure 4). Propoxur and bendiocarb are currently approved by WHOPES for IRS; carbofuran, carbaryl, aldicarb, and methomyl have been used as agricultural insecticides. Terbam has previously attracted our interest because of good toxicity to WT *An. gambiae* [21], [22]. Carbamates (C–X) are pseudo-irreversible inhibitors of AChE, that inactivate the enzyme by carbamoylation of the catalytic serine residue [33], [34]. Therefore, we used the Ellman Assay [27] to monitor time-dependent inhibition of the enzyme, by measuring enzyme velocities as a function of incubation time at fixed inhibitor concentrations. These velocities (*v*/*v*_0_) were used to calculate pseudo first-order rate constants *k*_obs_ (min^−1^) for inactivation by plotting ln(*v*/*v*_0_) vs incubation time *t*. For each inhibitor *k*_obs_ values were determined at three or more inhibitor concentrations ([I]). Plots of *k*_obs_ vs [I] were then constructed and the slope of the linear fit provided the apparent second-order rate constants *k*_i_ (mM^−1^ min^−1^) for inactivation (conversion of free enzyme E to carbamoylated enzyme E–C, Figure 4, Table 2). Such plots for propoxur and aldicarb are given in Figure 5A and B.

**Table 2:** Carbamate inactivation rate constants *k*_i_ for r*Ag*AChE (WT & G119S), *An. gambiae* homogenates (G3 & Akron), and r*h*AChE.^a^

Carbamate	r*Ag*AChE-WT *k*_i_ (mM^−1^ min^−1^)	*An. gambiae* G3 homogenate *k*_i_ (mM^−1^ min^−1^)^b^	r*Ag*AChE-G119S *k*_i_ (mM^−1^ min^−1^)	*An. gambiae* Akron homogenate *k*_i_ (mM^−1^ min^−1^)^c^	*rh*AChE *k*_i_ (mM^−1^ min^−1^)
propoxur	266±9	323±8	<0.037±0.007	<0.040±0.005	17.0±0.4
bendiocarb	839±22	865±41	<0.055±0.007	<0.053±0.008	111±5
carbofuran	2,620±150	2,760±110	<0.044±0.020	<0.069±0.010	428±12
carbaryl	386±10	343±8	<0.037±0.014	<0.049±0.015	15.4±0.4
terbam	1,510±100	1,710±20	0.40±0.03	0.65±0.06	126±3
aldicarb	13.3±0.3	13.6±0.3	3.15±0.08	2.94±0.04	6.5±0.3
methomyl	56.4±0.7	58.7±1.1	7.8±0.1	12.7±0.2	28.9±1.5
**4a**	498±13	521±37	10.7±0.1	12.7±0.4	79.8±2.4
**4b**	4,130±130	4,510±130	137±4	153±3	647±24
**4c**	9,140±260	10,400±400	290±7	305±9	805±36
**4d**	2,220±80	2,660±170	125±3	150±8	168±8
**4e**	2,380±50	2,760±50	36.5±0.8	29.8±0.9	174±7

a Measured at 23±1°C, pH 7.7, 0.1% (v/v) DMSO. ^b^G3 strain *An. gambiae* carry WT *Ag*AChE and possess a carbamate-susceptible phenotype. ^c^Akron strain *An. gambiae* carry G119S mutant *Ag*AChE and possess a carbamate-resistant phenotype.

As expected, *k*_i_ values at r*Ag*AChE-WT are very similar to those of *An. gambiae* G3 homogenate; *k*_i_ values at r*Ag*AChE-G119S are also very similar to those of *An gambiae* Akron homogenate (Table 2). Thus, the principal ATCh-hydrolyzing enzymes present in G3 and Akron homogenate appear to be the WT and G119S forms of *Ag*AChE, respectively. Inspection of Table 2 reveals that, as expected, carbamate inactivation of WT *Ag*AChE is much more rapid than that of the G119S resistant mutant; resistance ratios are given in Table 3. Resistance ratios for the carbamates bearing a benzene ring core (propoxur, bendiocarb, carbofuran, carbaryl and terbam) exceed 2,500, and the values obtained from recombinant enzymes closely match those obtained from homogenates. However, interestingly, resistance ratios for the oxime carbamates (aldicarb, methomyl) are less than 10, suggesting this structural motif is less affected by the G119S mutation. The greatly divergent resistance ratios of propoxur and aldicarb is visually discerned from the slopes of the WT and G119S *k*_obs_ vs [carbamate] plots in Figure 5A and B. Finally, as we have reported earlier, none of these commercial methylcarbamates offer appreciable selectivity for inhibition of WT *Ag*AChE over r*h*AChE [22].

**Table 3:** Enzyme resistance ratios and *Ag*AChE vs *h*AChE selectivity of selected carbamates.

Carbamate	*Ag*AChE source^a^	WT/G119S resistance ratio^b^	*Ag*/*h* selectivity^c^
propoxur	recombinant	7,200±1,400	16±1
	homogenate	8,100±1,000	19±1
bendiocarb	recombinant	15,000±2,000	7.6±0.4
	homogenate	16,000±3,000	7.8±0.5
carbofuran	recombinant	60,000±27,000	6.1±0.4
	homogenate	40,000±6,000	6.4±0.3
carbaryl	recombinant	10,000±4,000	25±1
	homogenate	7,000±2,000	22±1
terbam	recombinant	3,800±400	12±1
	homogenate	2,600±200	14±1
aldicarb	recombinant	4.2±0.1	2.0±0.1
	homogenate	4.6±0.1	2.1±0.1
methomyl	recombinant	7.2±0.1	2.0±0.1
	homogenate	4.6±0.1	2.0±0.1
**4a**	recombinant	47±1	6.2±0.2
	homogenate	41±3	6.5±0.5
**4b**	recombinant	30±1	6.4±0.3
	homogenate	30±1	7.0±0.3
**4c**	recombinant	32±1	11±1
	homogenate	34±2	13±1
**4d**	recombinant	18±1	13±1
	homogenate	18±1	16±1
**4e**	recombinant	65±2	14±1
	homogenate	93±3	16±1

a Recombinant sources of *Ag*AChE are r*Ag*AChE-WT and r*Ag*AChE-G119S; homogenates are G3 (WT) and Akron (G119S). ^b^Resistance ratio is calculated as *k*_i_(WT)/*k*_i_(G119S); values are taken from Table 2. Standard error in the ratio is calculated according to a standard propagation of error formula [61]. ^c^Selectivity for inhibiting *Ag*AChE (WT) vs *h*AChE, calculated as *k*_i_(*Ag*AChE)/*k*_i_(*h*AChE), with standard error in the ratio calculated according to a standard propagation of error formula [61].

### Toxicity of Aryl and Oxime Methylcarbamates to G3 and Akron Strain An. gambiae

Tarsal contact toxicity of these carbamates to live *An. gambiae* was then determined using the standard World Health Organization filter paper assay [35]. All compounds were toxic to G3 strain *An. gambiae*, with LC_50_ values ranging from 16 to 70 μg/mL (Table 4). As expected from the high resistance ratios seen at the enzyme level (2,600- to 60,000-fold), the benzene ring core carbamates (propoxur, bendiocarb, carbofuran, carbaryl and terbam) were much less toxic to Akron strain *An. gambiae*. Less than 10% Akron mortality at 24 h was seen for these compounds, at concentrations up to 5,000 μg/mL. However, aldicarb provides an important contrast, demonstrating similar high toxicities to Akron and G3 strains (LC_50_ values of 32 and 70 ug/mL respectively, Table 4). This result is consistent with the low resistance ratio seen at the enzyme level (4- to 5-fold). Yet curiously, methomyl was not appreciably toxic towards Akron strain despite the low (5- to 7-fold) resistance ratio at the enzyme. We believe that the divergent results for these two oxime carbamates may be related to the different consequences of oxidative metabolism of the two inhibitors, and offer further commentary in the Discussion. However, the low resistance ratios seen at the enzyme level for the oxime carbamates, and the excellent toxicity of aldicarb to Akron strain *An. gambiae* prompted us to explore other carbamate structures possessing core structures smaller than a 6-membered ring.

**Table 4:** Tarsal contact toxicity (24 h) to G3 and Akron strain *An. gambiae*, and toxicity resistance ratios.

Carbamate	*An. gambiae* G3 LC_50_ μg/mL (95% CI)	*An. gambiae* Akron LC_50_ μg/mL (95% CI)	Resistance ratio^c^
propoxur	39 (32–45)	>5,000^a^	>130
bendiocarb	16 (14–17)	>5,000^b^	>310
carbofuran	16 (11–25)	>5,000^b^	>310
carbaryl	42 (32–55)	>5,000^a^	>120
terbam	37 (14–60)	>5,000^a^	>130
aldicarb	70 (66–74)	32 (30–35)	0.5
methomyl	24 (17–37)	>5,000^a^	>200
**4a**	383 (355–420)	650 (488–859)	1.7
**4b**	96 (89–104)	81 (78–89)	0.8
**4c**	154 (140–167)	267 (241–289)	1.7
**4d**	138 (125–151)	231 (217–245)	1.7
**4e**	29 (26–32)	365 (344–384)	13

a No mortality at this concentration. ^b^Less than 10% mortality at this concentration. ^c^Defined by LC_50_ (Akron)/LC_50_ (G3).

### Synthesis and Evaluation of Pyrazol-4-yl Methylcarbamates

**Figure 6:**
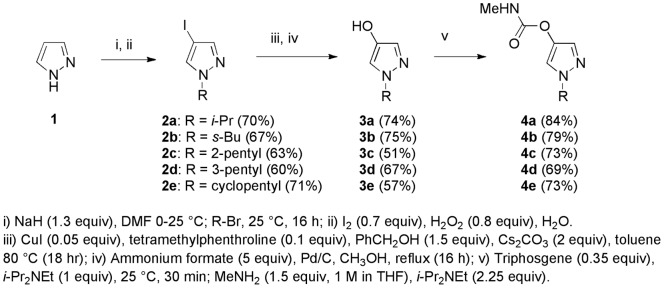
Synthesis of pyrazol-4-yl methylcarbamates 4a –**e.** All chiral compounds were prepared in racemic form. Detailed procedures are provided in on-line Supporting Materials (Text S1).

The G119S mutation should reduce the volume of the active site proximal to the oxyanion hole, since the small glycine side chain (H) is replaced by the hydroxymethyl group of serine [17], [23]. It thus seems likely that inhibitors occupying less volume in that region (e.g. aldicarb, methomyl) may be better able to carbamoylate the catalytic serine residue in the G119S mutant. To assess another class of “small-core” inhibitors we prepared a series of pyrazol-4-yl carbamates **4a**–**e**, as shown in Figure 6. The pyrazole ring was chosen in view of its slightly smaller size relative to benzene, and because its aromaticity should confer phenol-like character to the 4-hydroxypyrazole leaving group. *N*-Alkylation and iodination [36] of pyrazole **1** afforded intermediates **2a**–**e**. Subsequent copper-catalyzed benzyloxylation [37] and hydrogenolysis afforded 4-hydroxypyrazoles **3a**–**e**. Finally reaction with triphosgene and methylamine afforded the desired pyrazol-4-yl methylcarbamates **4a**–**e**. As hoped, these compounds exhibited potent inhibition of WT *Ag*AChE (recombinant and G3 homogenate), with *k*_i_ values ranging from 498 to 10,400 mM^−1^ min^−1^ (Table 2). Inhibition of G119S *Ag*AChE was slower, but the observed inactivation rate constants of 10.7 to 305 mM^−1^ min^−1^ are much greater than those of the aryl carbamates (Table 2), giving resistance ratios of only 18- to 65-fold (recombinant, Table 3). As indicated by the slopes of the lines in Figure 5A and C, inactivation of the G119S mutant by **4c** (290±7 mM^−1^ min^−1^) is even more rapid than inhibition of the WT enzyme by propoxur (266±9 mM^−1^ min^−1^). Most excitingly, these compounds, like aldicarb, proved toxic to Akron strain *An. gambiae*, exhibiting LC_50_ values of 81 to 650 μg/mL. The most toxic compound to Akron strain *An. gambiae* in this series (**4b**) was only 2- to 3-fold less toxic than aldicarb. Unfortunately, none of these compounds offer appreciable selectivity for inhibition of *Ag*AChE (WT or G119S) over *h*AChE (Table 3).

## Discussion

### Loss of Catalytic Efficiency in G119S AgAChE and Possible Compensatory Mechanisms in An. gambiae (Akron)

Although a number of *ace*-*1* resistance mutations have been identified for *Culex sp*. mosquitoes [24], [38], to date, G119S is the only *ace*-*1* resistance mutation characterized for *An. gambiae* [17], [23], [25]. MR4 reports that Akron strain *An. gambiae* are carbamate-resistant due to an *ace*-*1* mutation (www.mr4.org), but do not specify the identity of the mutation. By application of the published PCR-RFLP protocol [23], we established that Akron strain carries the G119S mutation and is homozygous resistant. As mentioned in the Results section, the WT catalytic domain construct of *Ag*AChE showed high specific activity (2,500±100 U/mg, Table 1). Interestingly, our measured *k*_cat_ value (1.8±0.1×10^5^ min^−1^) is very similar to that reported for WT *C. pipiens* AChE (1.9±0.2×10^5^ min^−1^); this latter value corresponds to a *V*_max_ of 3,100±300 U/mg, based on the reported subunit molecular mass of 60.4 kDa [38]. As we described above, the G119S mutant of *Ag*AChE suffers a major (34-fold) reduction in *k*_cat_, consistent with a significant change in the size of an active site residue side chain (−H to −CH_2_OH). This finding is similar to the 30-fold reduction in *k*_cat_ for G119S *C. pipiens* AChE relative to WT reported by Alout [38]. These dramatic reductions are not unexpected for an active site mutation, but find important precedent in previous work on the effect of oxyanion hole mutations in r*h*AChE [39]. This study showed that the G122A mutant of r*h*AChE, which corresponds to G119A in *Ag*AChE, suffered a 18-fold reduction in *k*_cat_ relative to WT r*h*AChE. Since the serine side chain is larger than that of alanine, it is not surprising that even larger reductions in *k*_cat_ were seen for the G119S mutants of *Ag*AChE and *C. pipiens* AChE. Also worthy of mention are earlier studies on the related enzyme human butyrylcholinesterase (*h*BChE), wherein replacement of the homologous residue G117 with histidine (i.e. G117H) led to a significant reduction in *k*_cat_ for butyrylthiocholine [40], [41].

The measured *K*_m_ value for our construct of recombinant WT *Ag*AChE (53.8±1.4 μM, Table 1) is similar to the 63.9±3.2 μM value reported by Jiang et al. for WT *Ag*AChE [28]. Thus in contrast to the dramatic effect seen on *k*_cat_, the G119S mutation causes only a 2-fold increase change in *K*_m_, both in the case of recombinant catalytic subunits and full-length native proteins in homogenate (Table 1). For comparison, the G122A mutation in *h*AChE increases *K*_m_ six-fold [39]. Why does the G119S mutation in *Ag*AChE (and G122A mutation in *h*AChE) affect *k*_cat_ more than *K*_m_? Since G119 is in the oxyanion hole [42], it is not directly involved in substrate binding. Instead, the NH group of this residue provides hydrogen-bond stabilization of the tetrahedral intermediate-like transition states on the reaction pathway from acetylthiocholine to thiocholine. The G119S mutation likely reduces the stabilization of one or more of these transition states, thereby significantly reducing turnover number (*k*_cat_).

In any event, the dramatic reduction seen in *k*_cat_ and *k*_cat_/*K*_m_ prompts the question of how G119S-AChE-bearing mosquitoes manage cholinergic neurotransmission. It is known that the G119S mutation reduces fitness in *C. pipiens* by a number of mechanisms, and increases mortality during pupation for *An. gambiae* [26]. Yet it seems likely that upregulation of AChE synthesis could provide a compensatory mechanism in adult *An. gambiae*. Our finding that Akron strain *An. gambiae* have 22% of the AChE activity of G3 strain *An gambiae* on a weight basis closely parallels the finding of Alout et al. [43], who reported that mosquito heads from Acerkis (G119S) strain *An. gambiae* had only 23% of the enzyme activity of heads from Kisumu (WT) strain *An. gambiae*. Based on our calculated *k*_cat_ values for *Ag*AChE-WT and *Ag*AChE-G119S, it appears that only an 8-fold increase in AChE concentration could account for the total AChE activity seen in Akron strain relative to G3 strain *An. gambiae*, and in Acerkis strain relative to Kisumu strain *An. gambiae*.

### Divergent Effects of G119 Mutations on Inhibition by Carbamates and Organophosphates; Consequences for Insecticide Resistance Mechanisms

Oxyanion hole mutations that significantly reduce *k*_cat_/*K*_m_ for substrate processing would well be expected to impart insensitivity towards acylation site inhibitors (i.e. carbamates and organophosphates). As this work and that of Alout et al. [43] have demonstrated, the G119S mutation in *Ag*AChE can dramatically reduce *k*_i_ values for some carbamate inhibitors, as was seen for G119S *Culex pipiens* AChE [44]. In addition, the G122A mutation of *h*AChE caused a greater than 50-fold decrease in *k*_i_ for inhibition by the carbamates physostigmine and pyridostigmine [39]. However the aforementioned G117H mutation of *h*BChE actually abolished organophosphate inhibition by introducing organophosphate hydrolase activity [40], [41]. This ability to turnover organophosphates was duplicated in a related triple mutant of *Bungarus fasciatus* AChE [45] and following mutation of the homologous oxyanion hole glycine of blowfly E3 carboxylesterase (G137D) [46]. The presence of the G137D mutation was subsequently characterized in an organophosphate resistant strain of blowfly [46]. Thus oxyanion hole mutations at G119 (*An. gambiae* numbering) can confer insensitivity (and thus resistance) to carbamates and organophosphates by at least two different mechanisms.

### Resistance Ratios: Comparison of Akron and Acerkis Strain An. gambiae

With respect to propoxur and aldicarb inactivation, our *k*_i_ values at WT (G3 homogenate) and G119S *Ag*AChE (Akron homogenate) can be compared to those of Alout et al., who reported *k*_i_ values for these carbamates using *An. gambiae* homogenates [43]. The Kisumu strain of *An. gambiae* was used as a source of WT *Ag*AChE, and their reported *k*_i_ values for propoxur (199±4 mM^−1^ min^−1^) and aldicarb (8.9±1.4 mM^−1^ min^−1^) are similar to our values from G3 homogenate (cf. Table 2). The Alout and Weill team used the Acerkis strain of *An. gambiae* as a source of G119S *Ag*AChE, and again their reported values for propoxur (0.002±0.0003 mM^−1^ min^−1^) and aldicarb (2.7±0.3 mM^−1^ min^−1^) are comparable to our values from Akron homogenate (cf. Table 2). Therefore, the Kisumu/Acerkis resistance ratios obtained by the Alout group for propoxur (∼100,000-fold) and aldicarb (3-fold) also match our findings (cf. Table 3). Thus the carbamate inhibition profiles of G3 *An. gambiae* follows that of the Kisumu strain; the same is true for the Akron and Acerkis strains. Finally, similar *k*_i_ values and resistance ratios for these two insecticides were also reported by Alout et al. for WT and G119S *Culex pipiens* [24], [44].

### Computational Modeling of Carbamate-AChE Interactions

**Figure 7:**
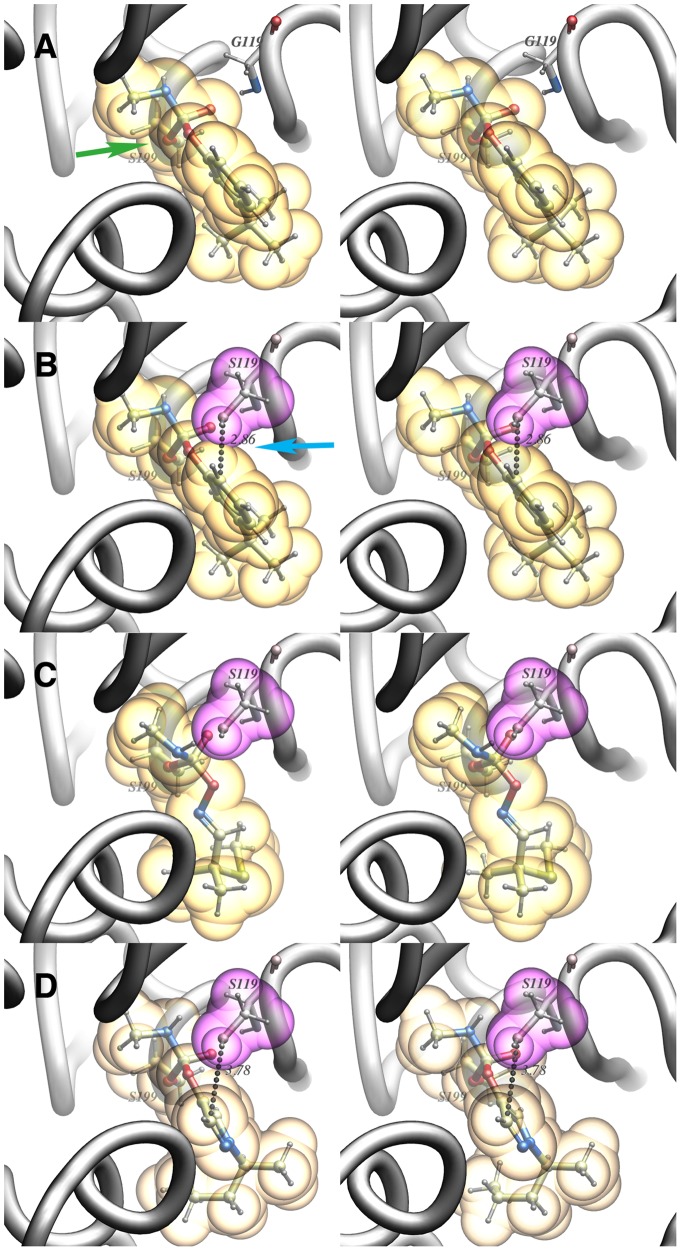
Computational modeling of tetrahedral intermediates formed by addition of the *Ag*AChE catalytic serine (S199) Oγ to carbamates. A) terbam with WT enzyme; location of catalytic serine oxygen Oγ is highlighted with the green arrow. B) terbam with G119S mutant enzyme; steric clash of the hydroxyl group of S119 with aromatic ring of terbam is noted with a blue arrow. C) Aldicarb with G119S mutant enzyme; note the absence of a steric clash with the hydroxyl group of S119. D) Pyrazol-4-yl methylcarbamate **4c** ((*S*)-enantiomer) with the G119S mutant enzyme; note the absence of a steric clash with the hydroxyl group of S119. Nonbonded contact distances in B and D are given in Å.

To gain structural insight into the high and low enzymatic resistance ratios indicated in Table 3, we computationally modeled the tetrahedral adducts of terbam with *Ag*AChE-WT and *Ag*AChE-G119S, and that of aldicarb and (*S*)-**4c** with *Ag*AChE-G119S. Note that both enantiomers of **4c** were sampled with this model, and the best-fitting enantiomer was chosen. The template for these four models was PDB ID 2H9Y, the tetrahedral adduct of mouse AChE and the trifluoromethylketone inhibitor TMTFA [47]. As can be seen in Figure 7, aromatic ring of terbam is well accommodated in the WT enzyme (Fig. 7A), but suffers steric repulsion with the S119 hydroxyl group in the G119S mutant enzyme (Figure 7B, blue arrow). However, since aldicarb and **4c** are smaller than terbam close to the carbamate carbonyl (cf. Figures 4 and 6), there is no apparent steric clash in the mutant enzyme tetrahedral adducts of aldicarb or **4c** (Fig. 7C, 7D). This admittedly preliminary modeling study is thus consistent with the low enzyme cross-resistance (4- and 34-fold, Table 3) seen for aldicarb and **4c**.

### Correlation of Akron Strain Toxicity to Rate of G119S AgAChE Inactivation

Toxicity to live WT and G119S AChE-bearing *An. gambiae* is a key criterion in the design of a new public health anticholinesterase insecticide. Our tarsal contact (filter paper) toxicity data for propoxur at G3 (WT) and Akron (G119S) strain *An. gambiae* closely match that reported by Djogbénou et al. for Kisumu (WT) and Acerkis (G119S) strain *An. gambiae* [25]. An important result from our work is the finding that aldicarb and pyrazol-4-yl methylcarbamates **4a**–**e** are very toxic to Akron strain *An. gambiae*, showing low toxicological cross-resistance (resistance ratios of 0.5- to 13-fold, Table 4). To our knowledge, this is the first published demonstration of good *in vivo* toxicities of carbamates to a carbamate-resistant phenotype mosquito, and suggests that other “small core” methylcarbamates could be developed to combat G119S-AChE-bearing vector mosquitoes. The low toxicological cross-resistance of these compounds is correlated to low enzymatic cross-resistance (4- to 65-fold, recombinant enzymes, Table 3). In contrast, aryl methylcarbamates show enzymatic cross-resistance of 4,000- to 60,000-fold (recombinant enzymes, Table 3), and toxicological cross-resistance exceeding 130-fold (note that in most cases, no Akron toxicity was observed at the highest dose tested). The one exception to this trend is methomyl, that is non-toxic to Akron *An. gambiae* at the highest concentration tested (5000 μg/mL), despite its low resistance ratio at the enzyme level (5- to 7-fold). We believe that some other factor, possibly oxidative metabolism, is an important determinant of the Akron toxicity of the two oxime carbamate insecticides. In addition to bearing the L1014F *kdr* mutation of the voltage-gated sodium ion channel, the MR4 Akron strain may feature significantly upregulated cytochrome P450 monooxygenases, such as those reported in other resistant strains of mosquitoes from southern Benin, including the Akron region [48]. In the case of aldicarb, increased oxidative metabolism could increase toxicity, since aldicarb sulfoxide is significantly more inhibitory to AChE [49], [50] and toxic to insects [50], [51] than is aldicarb itself. In contrast, the role of mixed function oxygenases in detoxifying methomyl is well known [52], [53], and thus it is possible that increased cytochrome P450 monooxygenases in Akron are responsible for the low methomyl toxicity observed.

### Conclusion

We have shown that potent inhibition of the G119S resistant mutant of *Ag*AChE, and high toxicity to *An. gambiae* carrying this mutation, is provided by “small-core” carbamates such as aldicarb and pyrazol-4-yl methylcarbamates **4a**–**e**. Although none of these compounds exhibit useful selectivity for inhibition of *Ag*AChE over *h*AChE, we have previously shown that appendage of the appropriate substituents to aryl carbamates can confer up to 500-fold selectivity [22]. Further modification of the *N*1-substituent of the pyrazol-4-yl methylcarbamates is in progress to achieve both *Anopheles* vs human-selectivity and resistance-breaking activity.

## Materials and Methods

### Materials

Recombinant *h*AChE (C1682) was purchased from Sigma-Aldrich (St. Louis, MO, USA). Based on the information provided by Sigma, the subunit molecular mass of commercial r*h*AChE is 64.70 kDa. Synthetic procedures and analytical characterization data for pyrazol-4-yl methylcarbamates **4a**–**e** are provided in the Text S1, as are the sources of commercial methylcarbamates and reagents.

### Mosquito Rearing, ace-1 Genotyping, and Preparation of Homogenates for Enzyme Assay

Both G3 and Akron strains of *An. gambiae* were obtained from MR4 (www.mr4.org) and have been in colony at Virginia Tech since 2005 (G3) and 2009 (Akron). The G3 strain (MRA-112, genotype 2La/+, 2r+/+, TEP1 s/s; phenotype red stripe, polymorphic for c+ (*collarless*)) is a “mongrel” strain and is reported to be sensitive to all insecticides [54]. The Akron strain (MRA-913, genotype M rDNA form, L1014F, *ace*-*1*; phenotype: carbamate resistant) originates from Porto Novo, Akron, Benin, and is reported by MR4 to have an *ace*-*1* mutation that confers the carbamate resistant phenotype [48]. To prevent cross-contamination, these strains were reared in separate environmental chambers (G3 28±1°C, 55–65% relative humidity (RH); Akron 28±1°C, 70% RH; both 14 h light, 10 h dark) using standard techniques. Pupae were removed daily to hatch in separate cages at 27±1°C and 80% RH, and adult mosquitoes were given free access to 10% (w/v) sugar water.

Genomic DNA was extracted from *An. gambiae* susceptible (G3) and resistant (Akron) strains. Individual, female mosquitoes were homogenized in Bender buffer containing 0.1 M NaCl, 0.2 M sucrose, 0.1 M Tris (pH 9.0), 0.05 M EDTA, and 0.5 M SDS. Mosquito homogenates were incubated overnight at 50°C with Proteinase K followed by phenol-chloroform extraction and ethanol precipitation. The PCR amplification of the G119/S119 region of *ace*-*1* was performed with Phusion™ *Taq* polymerase (New England Biolabs, Ipswich, MA), according to the manufacturer's instructions, with Moustdir1 (5′-CCGGGNGCSACYATGTGGAA-3′) and Moustrev1 (5′-ACGATMACGTTCTCYTCCGA-3′) degenerate primers (98°C, 1 min; 98°C, 10 sec; 61°C, 30 sec; 72°C, 10 sec; 35 cycles; 72°C, 10 min). The PCR amplicons were precipitated and digested for 16 h using *Alu*I restriction endonuclease (New England Biolabs) according to the manufacturer's instructions. The PCR amplicons were electrophoresed on a 1.3% agarose gel. The PCR amplicons were sequenced to confirm both the *Alu*I restriction site and G119S mutation. Nucleotide sequence alignments were generated by using Geneious Pro v5.4 [55].

For G3 homogenate, only female, non-blood fed, mosquitoes (>5 days old, live frozen) were used. Mosquito homogenates were prepared by combining 60 mosquitoes and 2.0 mL of ice-cold buffer (0.1 M sodium phosphate containing 0.02% NaN_3_ (w/v), 0.3% (v/v) Triton X-100, and 1 mg/mL bovine serum albumin (BSA), pH 7.7) in a tissue grinder for several seconds. The resulting suspensions were centrifuged at 2,400 g (30 min, 4°C). The supernatant was decanted into a passivated container (centrifuge tube or microcentrifuge tube pretreated with 5% Tween®-20 in MilliQ water, containing 0.02% NaN_3_ (w/v)) and stored overnight at 4°C. This stabilized supernatant could then be reliably used for enzymatic assay. Due to lower colony numbers, Akron homogenate was occasionally derived from both male and female mosquitoes (females not blood fed, both frozen live mosquitoes >5 days old). Separate control experiments established that Akron homogenate derived from male and non-blood fed female mosquitoes exhibited the same *K*_m_ value, within experimental error.

### AgAChE recombinant protein expression and purification

A forward primer (CTCGAGAAAAGAGAGGCTGACAACGATCCGCTGGTGGTCAA) and a reverse primer (TCTAGAGCTGCGCTGCTTTCGCACGGTT) containing an XhoI restriction site and an XbaI restriction site (underlined nucleotides), respectively, were designed and used for the amplification of the *Ag*AChE-WT (*ace*-*1*) catalytic domain. The amplified cDNA product was ligated into TA-cloning vector and then sub-cloned into a yeast protein expression vector (pPICZα A). The frame of the *Ag*AChE catalytic domain was verified by DNA sequencing. Another forward primer (GCTCTTCAAGTTTCTACTCCGGCACCGCCA) and reverse primer (GCTCTTCAACTGCCGCCG AAGATCCACAGCAT), both containing a SapI restriction site (underlined nucleotides), were also designed and paired with the 3′- and 5′- end primer, respectively to first amplify two separate DNA fragments, using the same sequencing verified *Ag*AChE-WT (*ace*-*1*) catalytic domain cDNA. Then, the two fragments were digested with SapI restriction enzyme, followed by ligation of the two fragments by T4 ligase, which produced a catalytic domain, identical to the wild-type, except for the change of the G119 to S119. The mutated catalytic domain was ligated into TA-cloning vector and then sub-cloned into the expression vector (pPICZα A), as was done for the WT catalytic domain. Note that residue numbering throughout this manuscript follows the catalytic subunit numbering convention resulting from alignment to D1 of *Torpedo californica* AChE [20]; to determine the full length numbering, add 161 to the residue number (cf. Swiss-Prot code ACES_ANOGA; *ace*-*1*, M1-Q737).

Recombinant pPICZα A vectors containing WT or G119S *Ag*AChE catalytic domains were linearized by BstXI and used to transform competent *Pichia pastoris* cells, based on the manufacturer's chemical transformation protocol (Invitrogen). Individual colonies were selected and tested for *Ag*AChE expression based on enzyme activity assays. The selected colony (showing high *Ag*AChE activity after methanol induction) was selected for large scale *Ag*AChE expression. These cells were cultured at 37°C and induced by methanol. After induction, the cells were cultured at 30°C for 48 hrs, broken down with glass beads, and centrifuged at 34,500 g (4°C, 30 min) [56]. The soluble proteins in the supernatant were applied to a column packed with nickel-chelating resin. After thorough washing with buffer, the recombinant proteins were eluted using a buffer containing 250 mM imidazole, 300 mM NaCl, and 50 mM sodium phosphate, pH 8.0. The affinity purification resulted in the isolation of each individual recombinant protein at about 70% purity. Further purifications of the recombinant proteins were achieved by Mono-Q and gel-filtration chromatographies. These purified proteins were concentrated to 5 mg protein/mL in 10 mM phosphate buffer (pH 7) using a Centricon YM-50 concentrator (Millipore). Purity of the recombinant proteins was evaluated by SDS-PAGE and the concentration of the purified recombinant proteins was determined by a Bio-Rad protein assay kit (Hercules, CA) using BSA as a standard. Expression yields of purified r*Ag*AChE (both WT and G119S) were approximately 0.25 mg/L.

### Enzyme Activity Measurements

Protein concentrations were determined using the Thermo Scientific Micro BCA^TM^ Protein Assay Kit (#23235) microplate procedure, and linear working range of 2–40 μg/ml. Enzyme velocities were measured in a microtiter plate format using the Ellman Assay [27]. Details are provided in Text S1.

### Determination of apparent second-order rate constants (k_i_) of for enzyme inactivation by carbamate inhibitors

Inhibition potency of carbamate insecticides was assessed by measuring apparent second-order rate constants *k*_i_ (mM^−1^ min^−1^) for inactivation of the enzymes. We adopted a progressive inactivation approach [33], [34], in which enzymes were incubated with different concentrations of carbamates for differing times before measuring enzyme residual activity (*v*/*v*_0_). Details are provided in Text S1.

### Computational Modeling of AgAChE/carbamate Interaction

An initial homology model of *Ag*AChE-WT was generated using the ICM homology modeling method [57], [58], [59], with the X-ray structure of mouse AChE complexed with the trifluoromethylketone ligand *m*-(*N*, *N*, *N*-trimethylammonio)trifluoroacetophenone (TMTFA; PDB code: 2H9Y) [47] as a template, as described previously [20]. This template was chosen because the covalent adduct of the catalytic serine with the TMTFA provided a close structural analogy to the tetrahedral adduct of carbamates (terbam and aldicarb) we intended to model. Furthermore, this structure (2.40 Å) offered superior resolution to the earlier 2.80 Å structure of TMTFA complexed to *Torpedo californica* AChE (PDB ID: 1AMN) [60]. A homology model of the resistant mutant was obtained by changing G119 to S119, and energy minimization. Covalent intermediate complexes of the carbamates were modeled by modifying the hydroxyl of the catalytic serine (S199) to the appropriate intermediate structures, and Monte-Carlo optimization of all torsions in the resulting modified side chains. The covalent intermediate complex of WT *Ag*AChE with terbam was superimposed with G119S mutant apo-structure to identify steric conflicts associated with the mutation. Next, the mutant G119S *Ag*AChE complexes with aldicarb and (*S*)-**4c** were modeled and analyzed to detect the presence or absence of similar steric conflicts. As mentioned above, for **4c** both enantiomers were sampled, and in this model, (*S*)-**4c** gives a better fit.

### Determination of carbamate toxicity to live An. gambiae

Adult female non-blood fed *An. gambiae* (both G3 and Akron strains) 3–5 days old, were used for filter paper assays of tarsal contact toxicity, which were performed in exposure tubes according to the 2006 World Health Organization recommendations [35], with slight modification, as described in Text S1.

## Supporting Information

Text S1 **Detailed methods and Additional Figures.** This document contains detailed experimental protocols, additional figures, synthetic procedures and analytical characterization data for pyrazol-4-yl methylcarbamates **4a**–**e**, and sourcing information for commercial inhibitors and reagents used in enzymatic assays.
(PDF)
