# Supplementary material for: Select Small Core Structure Carbamates Exhibit High Contact Toxicity to “Carbamate-Resistant” Strain Malaria Mosquitoes, Anopheles gambiae (Akron)
Source: PLoS One. 2012 Oct 1;7(10):e46712. doi: 10.1371/journal.pone.0046712 (PMC3462181; doi:10.1371/journal.pone.0046712)
Supplement: Text S1 — Detailed methods and Additional Figures. This document contains detailed experimental protocols, additional figures, synthetic procedures and analytical characterization data for pyrazol-4-yl methylcarbamates 4a–e, and sourcing information for commercial inhibitors and reagents used in enzymatic assays. (PDF) [file pone.0046712.s001.pdf]

TextS1: Supporting Information for the manuscript entitled

Select small core structure carbamates exhibit high contact toxicity to "carbamate-resistant" strain malaria mosquitoes, *Anopheles gambiae* (Akron)

Dawn M. Wong[a], Jianyong Li[b], Qiao-Hong Chen[a], Qian Han[b], James M. Mutunga[c], Ania Wysinski[c], Troy D. Anderson[c], Haizhen Ding[b], Tiffany L. Carpenetti[c], Astha Verma[a], Rafique Islam[d], Sally L. Paulson[c], Polo C.-H. Lam[e], Maxim Totrov[e], Jeffrey R. Bloomquist[c,d], Paul R. Carlier [a,*]

[a]Department of Chemistry, Virginia Tech, Blacksburg, VA 24061, USA
[b]Department of Biochemistry, Virginia Tech, Blacksburg, VA 24061, USA
[c]Department of Entomology, Virginia Tech, Blacksburg, VA 24061, USA
[d]Department of Entomology and Nematology, Emerging Pathogens Institute, University of Florida, Gainesville, FL 32610, USA
[e]Molsoft LLC, 11199 Sorrento Valley Rd, San Diego, CA, 92121, USA

*Corresponding author. Tel.: +1 540 231 9219; fax: +1 540 231 3255. E-mail address: pcarlier@vt.edu (P. R. Carlier)

Table of contents

Section	Description	Page
A.	Recombinant enzyme sequence and molecular weight	1
B.	pH profile of r*Ag*AChE-WT and r*Ag*AChE-G119S	2
C.	Michaelis-Menten plots of r*h*AChE, r*Ag*AChE-WT and r*Ag*AChE-G119S	2
D.	Commercial carbamates, reagents and buffers for enzyme assays	3
E.	Enzyme activity measurements	3
F.	Determination of apparent second-order rate constants (k_i) of for enzyme inactivation by carbamate inhibitors	4
G.	Determination of carbamate toxicity to live *An. gambiae*	4
H.	Synthetic procedures and analytical characterization for pyrazol-4-yl methylcarbamates	5
I.	References	8

A. Recombinant enzyme sequence and molecular weight

The gene sequence chosen gave a catalytic domain comprising D1 to S552, followed by a c-myc epitope, a spacer, and a 6xHis tag. Thus, the full sequence of the secreted protein should be D^1….S^{552}EQKLISEEDLNSAVEHHHHHH, comprising 573 aa. Calculated subunit molecular masses are 64.06 kDa (WT) and 64.09 kDa (G119S). Note that residue numbering throughout this manuscript will follow the catalytic subunit numbering convention resulting from alignment to D1 of *Torpedo californica* AChE [1]; to determine the full length numbering, add 161 to the residue number (cf. Swiss-Prot code ACES_ANOGA; *ace-1*, M1-Q737).

B. pH profile of r*Ag*AChE-WT and r*Ag*AChE-G119S

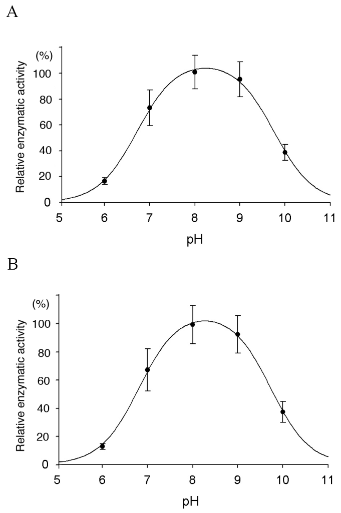

Figure S1. The pH dependence of *Ag*AChE velocity: **A**: r*Ag*AChE-WT; **B**: r*Ag*AChE-G119S mutant. Maximum velocity is seen near pH = 8.

C. Michaelis-Menten plots of r*h*AChE, r*Ag*AChE-WT and r*Ag*AChE-G119S

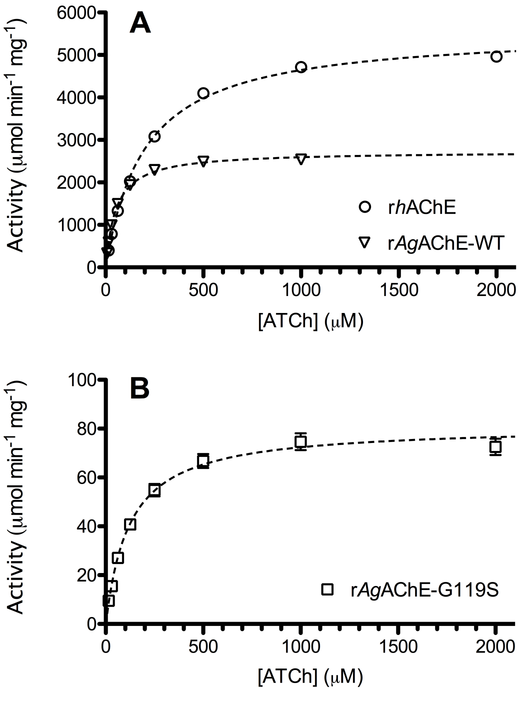

Figure S2. Michaelis-Menten plots for ATCh hydrolysis by A) r*h*AChE and r*Ag*AChE-WT, and B) Resistant mutant r*Ag*AChE-G119S.

D. Commercial carbamates, reagents and buffers for enzyme assays. The Ellman reagent (5,5'-dithiobis-(2-nitrobenzoic acid) (DTNB), ≥98%), acetylthiocholine iodide (ATCh, ≥98%), bovine serum albumin (BSA, heat shock fractionated, A7030, lyophilized powder, ≥98%), dimethyl sulfoxide (DMSO, ≥99.9%) and recombinant *h*AChE (C1682) were purchased from Sigma-Aldrich (St. Louis, MO, USA). Based on the information provided by Sigma, the subunit molecular mass of commercial r*h*AChE is 64.70 kDa. Aldicarb and methomyl were purchased from Chem Service, Inc., West Chester, PA, USA. Bendiocarb was purchased from Nor-Am Chemical Company, Wilmington, DE, USA. Propoxur (technical grade) was purchased from Mobay Chemical Corporation, Pittsburgh, PA, USA. Terbam was prepared in house at Virginia Tech [2]. Unless otherwise stated, the following buffers were used in this work: 1) buffer A is 0.1 M sodium phosphate buffer containing 0.02% NaN_3 (w/v), pH 7.7 at room temperature (23 ± 1°C), while 2) buffer B is buffer A containing 0.3% (v/v) Triton X-100, and 1 mg/mL bovine serum albumin (BSA), pH 7.7 at room temperature (23 ± 1°C).

E. Enzyme activity measurements

Protein concentrations were determined using the Thermo Scientific Micro BCA™ Protein Assay Kit (#23235) microplate procedure, and linear working range of 2-40 µg/ml. Wild-type enzymes (r*h*AChE, G3 homogenate, and r*Ag*AChE-WT) were diluted in ice-cold buffer B and observed in the Ellman assay (described below) to give an approximate reaction rate of 0.040 O.D./min (or ~0.0049 U/ml). Resistant enzymes (Akron homogenate and r*Ag*AChE-G119S) were diluted similarly and observed at a reaction rate of approximately 0.028 O.D./min (or 0.0034 U/ml). All diluted enzymes were kept over ice prior to use. Aliquots of the diluted enzymes (10 µl) thus prepared were separately incubated, in triplicate, in a 96-well microplate with buffer A (170 µl) for 10 min. Thereafter, a freshly prepared solution of ATCh and DTNB (4 and 3 mM respectively, in buffer A, 20 µl), was added and mixed manually to start the enzymatic reaction. Thus, a total volume of 200 µl and optical pathlength of 0.60 cm was achieved in each cell, with the following final concentrations: 0.015% (v/v) Triton X-100, 0.05 mg/ml BSA, 0.4 mM ATCh, 0.3 mM DTNB. Based on our protein concentration calculations, final enzyme concentrations employed in the activity and inhibition assays ranged from 29 to 36 pM (r*Ag*AChE-WT), 0.7 to 1.1 nM (r*Ag*AChE-G119S), and 18 to 23 pM (r*h*AChE). Enzyme activity was monitored continuously at 405 nm at room temperature (23 ± 1°C) for up to 3.4 min on a DYNEX Triad microplate reader (DYNEX Technologies, Chantilly, VA, USA), analyzed using the Concert TRIAD Series Analysis Software (version 2.1.0.17), corrected for spontaneous substrate hydrolysis. A molar extinction coefficient ε_{405} of 13,600 M^{-1} cm^{-1} was used for 2-mercapto-5-nitrobenzoic acid, the chromophore formed from the reaction of thiocholine and DTNB [3]. One unit (U) of AChE activity is defined as that amount of the enzyme that catalyzes the hydrolysis of 1 µmole of ATCh substrate per minute. For determination of k_{cat} and K_m, typically 8 concentrations of ATCh were employed (in duplicate). All V_{max} and K_m values were calculated by fitting the velocity (v) and substrate concentration ([S]) data to $v = V_{max}[S] / (K_m + [S])$ using Prism 5.0 (GraphPad Software Inc, La Jolla, CA, USA).

F. Determination of apparent second-order rate constants (k_i) of for enzyme inactivation by carbamate inhibitors.

Inhibition potency of carbamate insecticides was assessed by measuring apparent second-order rate constants k_i (mM^{-1} min^{-1}) for inactivation of the enzymes. We adopted a progressive inactivation approach [4,5], in which enzymes were incubated with different concentrations of carbamates for differing times before measuring enzyme residual activity (v/v_0). The plate loading protocol described in Section E above was used with slight modification. Instead of adding 170 μL of buffer A, 150 μL of buffer A and 20 μL of an inhibitor solution was added. The inhibitor solutions were prepared in buffer A with a DMSO concentration of 1% (v/v); the final assay concentration of DMSO was thus 0.1% (v/v). After waiting for the desired incubation time *t*, substrate and indicator were added as described in Section E above, and the enzymatic reaction was followed at 405 nm. As described above, enzymes were incubated with typically five concentrations of inhibitors (and an inhibitor-free control) for up to 6 minutes at approximately 1 min intervals. Each concentration was present in the microplate in duplicate, and each experiment was repeated. Note that for the G119S enzymes (recombinant and Akron homogenate) inhibition was typically very low after 10 min at 10 μM, and so incubations with these enzymes were often extended to 60 min (10 min intervals). Residual activities v/v_0 are the ratio of the rate in the presence of inhibitor to a time-matched inhibitor-free control. This method therefore corrects for slow thermal inactivation of the enzyme. As described in the Results Section of this manuscript, these residual activities were used to calculate pseudo first-order rate constants k_{obs} (in duplicate) for inactivation at three or more inhibitor concentrations ([I]). Plots of k_{obs} vs [I] were then constructed as shown in Figure 5; in each case the slope of the unconstrained linear fit is the apparent second-order rate constant k_i (mM^{-1} min^{-1}) for inactivation. The error in k_i is estimated as the standard error in the slope of this plot. Note that inhibitor concentrations [I] were chosen to be low enough to remain in the domain where a plot of k_{obs} vs [I] was linear. For fast-inactivating carbamates, such as terbam on r*Ag*AChE-WT or G3 homogenate, we saw signs of saturation behavior above [I] = 0.2 μM. Saturation behavior is expected for a two-step mechanism of inhibition, such as that depicted in Figure 4 [6]. Resistance ratios and *Ag*/*h* selectivity for inhibition (Table 3) were calculated from the measured k_i values; the error in these ratios was determined using a standard propagation of error method [7].

G. Determination of carbamate toxicity to live *An. gambiae*

Adult female non-blood fed *An. gambiae* (both G3 and Akron strains) 3-5 days old, were used for filter paper assays of toxicity, which were performed in exposure tubes according to the 2006 World Health Organization recommendations, with slight modification [8]. In brief, filter papers (15 x 12 cm) were treated with 2.0 mL of various concentrations of the carbamate in ethanol, and allowed to dry overnight. For the G3 strain, batches of 20-25 mosquitoes (in triplicate) were transferred to a holding tube and allowed to adapt for one hour. Due to lower colony numbers, toxicity measurements with Akron strain used batches of 10-15 mosquitoes in duplicate. Mosquitoes were then transferred to the exposure tube (held horizontally) that contained a treated filter paper. Knockdown was noted after 1 h, and all mosquitoes were transferred back to the holding tube (held upright), and given free access to 10% (w/v) sugar water. Mortality was recorded at 24 h. Both during exposure and the 23 h period following, mosquito tubes were kept in an environmental chamber at 24 ± 1 °C and 75% RH. To determine LC_{50} values, typically 5-8

concentrations were examined, and mortality data were used for probit analysis using PoloPlus [9].

H. Synthetic procedures and analytical characterization for pyrazol-4-yl methylcarbamates

Analytical and purification protocols.
NMR spectra were obtained at 500 (^{1}H) and 126 (^{13}C) MHz. The chemical shifts are given in δ (ppm), and coupling constants are reported in Hz. High-resolution ESI Mass spectra were obtained on an Agilent 6220 Accurate Mass TOF LC/MS. THF was distilled from sodium-benzophenone and dichloromethane was distilled from calcium hydride. Other reagents and solvents were purchased from commercial sources and were used without further purification. Silica gel column chromatography was performed using flash silica gel (32-63 μ). Preparative thin-layer chromatography (PTLC) separations were carried out on 1000 μ Uniplate thin layer chromatography plates.

General procedure for the synthesis of 1-alkyl-4-iodopyrazoles (2a-e): To a mixture of sodium hydride (60% dispersion in mineral oil, 520 mg, 13 mmol) in DMF (4 mL) at 0 °C was added dropwise a solution of pyrazole (680 mg, 10 mmol) in DMF (1 mL), and the subsequent mixture was kept stirring at room temperature for 1 h. To this mixture was added alkyl bromide (15 mmol), and the reaction was allowed to proceed at room temperature (25 °C) for 16 h. The reaction mixture was diluted with diethyl ether, which was rinsed with brine. After removing the diethyl ether, the crude product was directly used for the next step reaction without further purification. To a mixture of 1-alkylpyrazole (nominally 10 mmol) in water (7 mL) was sequentially added iodine (7 mmol) and 35% hydrogen peroxide (8.4 mmol), and the mixture was kept stirring for 24 h at room temperature. The reaction was quenched by the addition of a cold solution of saturated sodium thiosulfate (60 mL), and the subsequent mixture was extracted with ethyl acetate (3 x 50 mL). The combined extracts were dried over anhydrous sodium sulfate and concentrated *in vacuo*. The residue obtained was chromatographed over silica gel eluting with 5% ethyl acetate in hexanes to furnish the respective 1-alkyl-4-iodopyrazole (**2a**-**e**).

4-Iodo-1-isopropyl-1*H*-pyrazole (2a): This compound was prepared as a colorless oil from the crude product of 1-isopropyl-1*H*-pyrazole in 70% overall yield for two steps. ^{1}H NMR (500 MHz, $CDCl_3$) *d* 7.47 (s, 1H), 7.43 (s, 1H), 4.47 (heptet, *J* = 6.7 Hz, 1H), 1.46 (d, *J* = 6.7 Hz, 6H); ^{13}C NMR (125 MHz, $CDCl_3$) *d* 143.8, 131.1, 55.4, 54.5, 22.9. This compound has been described previously [10].

1-*sec*-Butyl-4-iodo-1*H*-pyrazole (2b): This compound was prepared as a colorless oil from the crude product of 1-*sec*-butyl-1*H*-pyrazole in 67% overall yield for two steps. ^{1}H NMR (500 MHz, $CDCl_3$) *d* 7.49 (s, 1H), 7.42 (s, 1H), 4.21 (hextet, *J* = 6.8 Hz, 1H), 1.82-1.90 (m, 1H), 1.71-1.79 (m, 1H), 1.46 (d, *J* = 6.8 Hz, 3H), 0.80 (t, *J* = 7.4 Hz, 3H); ^{13}C NMR (125 MHz, $CDCl_3$) *d* 143.8, 131.8, 60.6, 55.3, 30.2, 20.9, 10.6. This compound is described by Chemical Abstracts Service as Registry Number 1339666-74-8.

4-Iodo-1-(pentan-2-yl)-1*H*-pyrazole (2c): This compound was prepared from the crude product of 1-(pentan-2-yl)-1*H*-pyrazole in 63% overall yield for two steps. Pale yellow oil; ^{1}H NMR

(500 MHz, $CDCl_3$) *d* 7.48 (s, 1H), 7.41 (s, 1H), 4.27-4.33 (m, 1H), 1.81-1.88 (m, 1H), 1.63-1.70 (m, 1H), 1.45 (d, J = 6.8 Hz, 3H), 1.10-1.25 (m, 2H), 0.87 (t, J = 7.4 Hz, 3H); ^{13}C NMR (125 MHz, $CDCl_3$) *d* 143.8, 131.8, 58.9, 55.3, 39.2, 21.4, 19.3, 13.7; HRMS (ESI) calcd for $C_8H_{13}N_2I$ [M+H] 265.0116, found 265.0189.

4-Iodo-1-(pentan-3-yl)-1*H*-pyrazole (2d): This compound was prepared from the crude product of 1-(pentan-3-yl)-1*H*-pyrazole in 60% overall yield for two steps. Pale yellow oil; ^1H NMR (500 MHz, $CDCl_3$) *d* 7.50 (s, 1H), 7.39 (s, 1H), 3.85-3.91 (m, 1H), 1.74-1.87 (m, 4H), 0.74 (t, J = 7.4 Hz, 6H); ^{13}C NMR (125 MHz, $CDCl_3$) *d* 144.0, 132.8, 67.2, 55.2, 28.5, 10.7; HRMS (ESI) calcd for $C_8H_{13}N_2I$ [M+H] 265.0126, found 265.0199.

1-Cyclopentyl-4-iodo-1*H*-pyrazole (2e): This compound was prepared as a colorless oil from the crude product of 1-cyclopentyl-1*H*-pyrazole in 71% overall yield for two steps. ^1H NMR (500 MHz, $CDCl_3$) *d* 7.48 (s, 1H), 7.44 (s, 1H), 4.63 (quintet, J = 6.8 Hz, 1H), 2.10-2.17 (m, 2H), 1.93-2.01 (m, 2H), 1.80-1.88 (m, 2H), 1.64-1.74 (m, 2H); ^{13}C NMR (125 MHz, $CDCl_3$) *d* 143.8, 132.0, 63.5, 53.4, 33.0, 24.1. This compound is described by Chemical Abstracts Service as Registry Number 1194377-14-4.

General procedure for the synthesis of 1-alkyl-4-hydroxypyrazoles (3a-e): A thick-walled sealable reaction tube was charged with CuI (9.5 mg, 0.05 mmol), 3,4,7,8-tetramethyl-1,10-phenantholine (24 mg, 0.10 mmol), Cs_2CO_3 (490 mg, 1.5 mmol), *N*-alkyl-4-iodopyrazole (1.0 mmol), benzyl alcohol (0.16 mL, 1.5 mmol), toluene (0.50 mL), and a magnetic stir bar. The was purged with dry nitrogen and then quickly sealed with a teflon screwcap. The vessel was immersed in a 80 °C oil bath and stirred vigorously for 18 h. The reaction mixture was allowed to cool to room temperature, diluted with ethyl acetate (15 mL), filtered through a plug of silica gel, and further eluted with additional ethyl acetate (30 mL). The filtrate was concentrated and the resulting residue was purified by column chromatography over silica gel (10% ethyl acetate in hexanes) to provide the desired 1-alkyl-4-benzyloxypyrazole. This material (nominally 1 mmol) was then dissolved in methanol (7 mL), to which was sequentially added 10% Pd-C (50% wet, 230 mg) and ammonium formate (315 mg, 5 mmol), and the reaction was allowed to proceed under reflux for 16 h. After cooling to room temperature the reaction was filtered through a silica gel pad, which was then rinsed with 100 mL of ethyl acetate. The filtrate was evaporated under reduced pressure to afford the desired products **3a-e**. The physical and spectroscopic data of the 1-alkyl-4-hydroxypyrazoles are listed below:

1-Isopropyl-1*H*-pyrazol-4-ol (3a): This compound was prepared from 4-iodo-1-isopropyl-1*H*-pyrazole (**2a**) in 74% overall yield for two steps. Colorless oil; ^1H NMR (500 MHz, $CDCl_3$) *d* 8.58 (br.s, 1H), 7.10 (d, J = 0.8 Hz, 1H), 7.08 (d, J = 0.8 Hz, 1H), 4.36 (heptet, J = 6.8 Hz, 1H), 1.41 (d, J = 6.8 Hz, 6H); ^{13}C NMR (125 MHz, $CDCl_3$) *d* 141.5, 127.2, 114.0, 54.1, 22.8. This compound has been described previously [10].

1-*sec*-Butyl-1*H*-pyrazol-4-ol (3b): This compound was prepared from 1-*sec*-butyl-4-iodo-1*H*-pyrazole **(2b)** in 75% overall yield for two steps as a pale yellow oil. ^1H NMR (500 MHz, $CDCl_3$) *d* 7.15 (s, 1H), 7.07 (s, 1H), 4.03-4.11 (m, 1H), 1.76-1.83 (m, 1H), 1.67-1.74 (m, 1H), 1.41 (d, J = 6.8 Hz, 3H), 0.78 (t, J = 7.4 Hz, 3H); ^{13}C NMR (125 MHz, $CDCl_3$) *d* 141.0, 127.5, 114.7, 60.3, 30.2, 20.9, 10.7. This compound has been described previously [10].

1-(Pentan-2-yl)-1*H*-pyrazol-4-ol (3c): This compound was prepared from 4-iodo-1-(pentan-2-yl)-1*H*-pyrazole **(2c)** in 51% overall yield for two steps as a pale yellow oil. ^{1}H NMR (500 MHz, $CDCl_3$) *d* 7.13 (s, 1H), 7.06 (s, 1H), 4.12-4.19 (m, 1H), 1.73-1.82 (m, 1H), 1.58-1.66 (m, 1H), 1.40 (d, *J* = 6.8 Hz, 3H), 1.09-1.23 (m, 2H), 0.85 (t, *J* = 7.4 Hz, 3H); ^{13}C NMR (125 MHz, $CDCl_3$) *d* 141.4, 127.1, 114.7, 58.6, 39.3, 21.3, 19.4, 13.7; HRMS (ESI) calcd for $C_8H_{14}N_2O$ [M+H] 155.1113, found 155.1186.

1-(Pentan-3-yl)-1*H*-pyrazol-4-ol (3d): This compound was prepared from 4-iodo-1-(pentan-3-yl)-1*H*-pyrazole **(2d)** in 67% overall yield for two steps as a pale yellow oil. ^{1}H NMR (500 MHz, $CDCl_3$) *d* 8.29 (br.s, 1H), 7.13 (s, 1H), 7.02 (s, 1H), 3.71-3.77 (m, 1H), 1.69-1.77 (m, 4H), 0.72 (t, *J* = 7.2 Hz, 6H); ^{13}C NMR (125 MHz, $CDCl_3$) *d* 141.3, 127.3, 115.4, 66.9, 28.5, 10.7; HRMS (ESI) calcd for $C_8H_{14}N_2O$ [M+H] 155.1104, found 155.1177.

1-Cyclopentyl-1*H*-pyrazol-4-ol (3e): This compound was prepared from 4-iodo-1-cyclopentyl-1*H*-pyrazole **(2e)** in 57% overall yield for two steps as a pale yellow solid. ^{1}H NMR (500 MHz, $CDCl_3$) *d* 8.44 (br.s, 1H), 7.09 (s, 1H), 7.07 (s, 1H), 4.49 (quintet, *J* = 7.0 Hz, 1H), 2.05-2.13 (m, 2H), 1.74-1.90 (m, 4H), 1.57-1.67 (m, 2H); ^{13}C NMR (125 MHz, $CDCl_3$) ***d*** 141.1, 127.6, 115.0, 63.3, 32.8, 24.0. This compound has been described previously [10].

General procedure for the synthesis of 1-alkylpyrazol-4-yl methylcarbamates (4a-e): This procedure is loosely based on the 7-hydroxycoumarin alanine methyl ester carbamate protocol of Seto [11]. To a solution of triphosgene (99 mg, 0.33 mmol, 0.33 equiv) in dichloromethane (10 mL) was slowly added a solution of 1-alkyl-4-hydroxypyrazole (1 mmol, 1 equiv) and diisopropylethylamine (1 mmol, 1 equiv) in THF (10 mL), and the mixture was stirred at room temperature for 30 min. To the reaction mixture, a solution of methylamine (2M in THF, 1.5 mmol, 1.5 equiv) and diisopropylethylamine (2.25 mmol, 2.25 equiv) in dichloromethane (2 mL) was added, and the reaction was allowed to proceed for an additional 30 min. The subsequent reaction mixture was partitioned between dichloromethane and water, and the organic phase was dried over anhydrous sodium sulfate and concentrated. The residue obtained was purified by column chromatography eluting with 20% ethyl acetate in hexanes to give the respective carbamate. The physical and spectroscopic data of the carbamates are listed below:

1-Isopropyl-1*H*-pyrazol-4-yl methylcarbamate (4a): yield: 84%; pale yellow oil; ^{1}H NMR (500 MHz, $CDCl_3$) *d* 7.51 (s, 1H), 7.35 (s, 1H), 5.03 (br.s, 1H), 4.39 (heptet, *J* = 6.7 Hz, 1H), 2.86 (d, *J* = 4.9 Hz, 3H), 1.46 (d, *J* = 6.7 Hz, 6H); ^{13}C NMR (125 MHz, $CDCl_3$) *d* 154.6, 135.3, 129.5, 117.4, 54.4, 27.8, 22.8; HRMS (ESI) calcd for $C_8H_{14}N_3O_2$ [M+H] 184.1081, found 184.1094. This compound has been described previously [10].

1-*sec*-Butyl-1*H*-pyrazol-4-yl methylcarbamate (4b): yields: 79%; pale yellow oil; ^{1}H NMR (500 MHz, $CDCl_3$) *d* 7.49 (s, 1H), 7.35 (s, 1H), 5.04 (br.s, 1H), 4.10 (hextet, *J* = 7.1 Hz, 1H), 2.85 (d, *J* = 4.9 Hz, 3H), 1.80-1.88 (m, 1H), 1.68-1.77 (m, 1H), 1.44 (d, *J* = 6.8 Hz, 3H), 0.79 (t, *J* = 7.4 Hz, 3H); ^{13}C NMR (125 MHz, $CDCl_3$) *d* 154.6, 135.3, 129.4, 118.1, 60.5, 30.2, 27.8, 20.8, 10.6; HRMS (ESI) calcd for $C_9H_{16}N_3O_2$ [M+H] 199.1265, found 199.1294. This compound has been described previously [10].

1-(Pentan-2-yl)-1*H*-pyrazol-4-yl methylcarbamate (4c): yield: 73%; colorless oil; ^{1}H NMR (500 MHz, $CDCl_3$) *d* 7.50 (s, 1H), 7.35 (s, 1H), 4.93 (br.s, 1H), 4.21 (hexet, *J* = 6.8 Hz, 1H), 2.87 (d, *J* = 4.9 Hz, 3H), 1.80-1.87 (m, 1H), 1.61-1.68 (m, 1H), 1.44 (d, *J* = 6.8 Hz, 3H), 1.12-1.27 (m, 2H), 0.87 (t, *J* = 7.2 Hz, 3H); ^{13}C NMR (125 MHz, $CDCl_3$) *d* 154.6, 135.3, 129.4, 118.0, 58.8, 39.3, 27.8, 21.1, 19.4, 13.8; HRMS (ESI) calcd for $C_{10}H_{18}N_3O_2$ [M+H] 212.1394, found 212.1396; calcd for $C_{10}H_{17}N_3NaO_2$ [M+Na] 234.1213, found 234.1215; calcd for $C_{10}H_{17}KN_3O_2$ [M+K] 250.0952, found 250.0955.

1-(Pentan-3-yl)-1*H*-pyrazol-4-yl methylcarbamate (4d): yield: 69%; pale yellow oil; ^{1}H NMR (500 MHz, $CDCl_3$) *d* 7.48 (s, 1H), 7.37 (s, 1H), 5.10 (br.s, 1H), 3.76-3.83 (m, 1H), 2.85 (d, *J* = 4.9 Hz, 3H), 1.72-1.87 (m, 4H), 0.76 (t, *J* = 7.4 Hz, 6H); ^{13}C NMR (125 MHz, $CDCl_3$) *d* 154.6, 135.2, 129.5, 118.9, 67.2, 28.5, 27.8, 10.8; HRMS (ESI) calcd for $C_{10}H_{18}N_3O_2$ [M+H] 212.1394, found 212.1400; calcd for $C_{10}H_{17}N_3NaO_2$ [M+Na] 234.1213, found 234.1222; calcd for $C_{10}H_{17}KN_3O_2$[M+K] 250.0952, found 250.0963.

1-Cyclopentyl-1*H*-pyrazol-4-yl methylcarbamate (4e): yield: 73%; white solid; ^{1}H NMR (500 MHz, $CDCl_3$) *d* 7.52 (s, 1H), 7.36 (s, 1H), 4.93 (br.s, 1H), 4.56 (quintet, *J* = 7.1 Hz, 1H), 2.87 (d, *J* = 5.0 Hz, 3H), 2.09-2.16 (m, 2H), 1.94-2.01 (m, 2H), 1.79-1.87 (m, 2H), 1.62-1.71 (m, 2H); ^{13}C NMR (125 MHz, $CDCl_3$) *d* 154.6, 135.3, 129.7, 118.5, 63.6, 32.9, 27.8, 24.2; HRMS (ESI) calcd for $C_{10}H_{16}N_3O_2$ [M+H] 210.1237, found 210.1241. This compound has been described previously [10].

I. References

1. Carlier PR, Anderson TD, Wong DM, Hsu DC, Hartsel J, et al. (2008) Towards a species-selective acetylcholinesterase inhibitor of the mosquito vector of malaria, *Anopheles gambiae*. Chemico-Biological Interactions 175: 368-375.
2. Hartsel JA, Wong DM, Mutunga JM, Ma M, Anderson TD, et al. (2012) Re-engineering aryl methylcarbamates to confer high selectivity for inhibition of *Anopheles gambiae* versus human acetylcholinesterase. Bioorganic & Medicinal Chemistry Letters, 22: 4593-4598.
3. Ellman GL, Courtney KD, Andres VJ, Featherstone RM (1961) A new and rapid colorimetric determination of acetylcholinesterase activity. Biochemical Pharmacology 7: 88-95.
4. Bar-On P, Millard CB, Harel M, Dvir H, Enz A, et al. (2002) Kinetic and structural studies on the interaction of cholinesterases with the anti-Alzheimer drug rivastigmine. Biochemistry 41: 3555-3564.
5. Reiner E, Aldridge WN (1967) Effect of pH on Inhibition and Spontaneous Reactivation of Acetylcholinesterase Treated with Esters of Phosphoric Acids and of Carbamic Acids. Biochemical Journal 105: 171-179.
6. Copeland RA (2005) Irreversible Enzyme Inactivators. Evaluation of Enzyme Inhibitors in Drug Discovery. New York: Wiley-Interscience. pp. 214-219.
7. Andraos J (1996) On the Propagation of Statistical Errors for a Function of Several Variables. Journal of Chemical Education 73: 150-154.
8. World Health Organization 2006. Guidelines for testing mosquito adulticides for indoor residual spraying and treatment of mosquito nets. Document WHO/CDS/NTD/WHOPES/GCDPP/2006.3, Geneva.

9. Robertson, J.L, H.K. Preisler, and R. M. Russell. 2002. PoloPlus Probit and Logit Analysis. LeOra Software.
10. 4-Pyrazolyl N-methylcarbamates. Maurer F, Hammann, I; Homeyer B; Behrenz W. Eur. Pat. Appl (1981), EP 23326 A1 19810204.
11. Xue F, Seto CT (2010) Fluorogenic Peptide Substrates for Serine and Threonine Phosphatases. Organic Letters 12: 1936-1939.
